# Evaluating the Impact of Wildlife Shelter Management on the Genetic Diversity of *Erinaceus europaeus* and *E. roumanicus* in Their Contact Zone

**DOI:** 10.3390/ani10091452

**Published:** 2020-08-20

**Authors:** Kerstin Ploi, Manuel Curto, Barbora Černá Bolfíková, Miroslava Loudová, Pavel Hulva, Anna Seiter, Marilene Fuhrmann, Silvia Winter, Harald Meimberg

**Affiliations:** 1 Institute for Integrative Nature Conservation Research, University of Natural Resources and Life Sciences (BOKU), 1180 Vienna, Austria; kerstin.ploi@students.boku.ac.at (K.P.); manuel.curto@boku.ac.at (M.C.); anna.seiter@posteo.de (A.S.); marilene.fuhrmann@students.boku.ac.at (M.F.); 2 MARE–Marine and Environmental Sciences Centre, Universidade de Lisboa, 1649-004 Lisboa, Portugal; 3 Faculty of Tropical AgriSciences, Czech University of Life Sciences Prague, Kamýcká 129, 165 21 Prague, Czech Republic; bolfikova@ftz.czu.cz; 4 Department of Zoology, Faculty of Science, Charles University in Prague, Viničná 7, 116 36 Prague, Czech Republic; miroslava.loudova@natur.cuni.cz (M.L.); pavel.hulva@natur.cuni.cz (P.H.); 5 Faculty of Science, University of Ostrava, Chittussiho 10, 710 00 Ostrava, Czech Republic; 6 Institute of Plant Protection, University of Natural Resources and Life Sciences (BOKU), 1180 Vienna, Austria; silvia.winter@boku.ac.at

**Keywords:** hedgehog, animal shelter, translocation, genotyping by amplicon sequencing

## Abstract

**Simple Summary:**

Hedgehogs are regularly brought to wildlife shelters. Depending on the area from where animals are accepted, translocation can occur between different regions or populations. In this study, the genetic diversity of wild hedgehog populations was compared with “shelter populations” within central Europe focusing on the western contact zone between both European hedgehog species. Some shelters were hosting both species at the same time, in one this could be shown genetically. Generally, no difference in genetic diversity between shelter individuals and wild populations was found. Two shelters from Innsbruck hosted individuals that probably belong to two subpopulations. This indicates that shelter management-related translocations could facilitate gene flow across a dispersal barrier.

**Abstract:**

Hedgehogs are among the most abundant species to be found within wildlife shelters and after successful rehabilitation they are frequently translocated. The effects and potential impact of these translocations on gene flow within wild populations are largely unknown. In this study, different wild hedgehog populations were compared with artificially created “shelter populations”, with regard to their genetic diversity, in order to establish basic data for future inferences on the genetic impact of hedgehog translocations. Observed populations are located within central Europe, including the species *Erinaceus europaeus* and *E. roumanicus*. Shelters were mainly hosting one species; in one case, both species were present syntopically. Apart from one exception, the results did not show a higher genetic diversity within shelter populations, indicating that individuals did not originate from a wider geographical area than individuals grouped into one of the wild populations. Two shelters from Innsbruck hosted individuals that belonged to two potential clusters, as indicated in a distance analysis. When such a structure stems from the effects of landscape elements like large rivers, the shelter management-related translocations might lead to homogenization across the dispersal barrier.

## 1. Introduction

The two European hedgehog species *Erinaceus europaeus* (Linne, 1758) and *E. roumanicus* (Barrett-Hamilton, 1900) are assessed as ‘least concern’ by the IUCN (International Union for Conservation of Nature) Red List of Threatened Species [1,2]. Nevertheless, in several western European countries like England, the Netherlands, and Belgium, road-kill and citizen science data suggested a decline of *E. europaeus* [3,4,5,6]. Investigations on the hedgehog population structure have been performed across a wide range of European countries [7,8], including their central European zone of sympatry and potential hybridization zone [8,9,10]. Because hedgehogs are mainly found in close proximity to human settlements, rural as well as urban, occupying diverse man-made habitats [1,2,11], the population structure might be influenced by human infrastructure and anthropogenic barriers (e.g., roads and major transportation axes) fragmenting the landscape. Limited migration possibilities might consequently reduce and disturb gene flow [4,12,13,14]. This can manifest itself in an influence on the isolation-by-distance pattern on small geographical scales, like shown in the United Kingdom (UK) [7]. As small mammal species, hedgehogs are naturally subjected to dispersal impediments that can result in a genetic structure [15,16]. Therefore, the effects of anthropogenic as well as geographical barriers (e.g., rivers, mountain ranges) might be reinforced by the per se limited dispersal capacities of this species [7,9,14].

As likeable and well-known garden species, hedgehogs are often used as flagship or umbrella species in urban nature conservation (e.g., the garden projects “Garten Charata” in Switzerland, “Natur im Garten” in Austria, “Super-Igel-Garten, NAJU Rostock” in Germany). Due to this medial presence, and their popularity and presence in gardens, they are often the center of attention when it comes to human-mediated rescue actions. Hedgehogs are frequently translocated and among one of the most abundant species found in wildlife shelters in western Europe [17]. Wildlife shelters rehabilitating hedgehogs can also be found across Austria (at least one in every federal province) [18]. The primary aim of these shelters lies in the successful rehabilitation and subsequent release of their temporary patients back into the wild. This, however, may result in (unintentional) translocation; if the individual’s origin is unknown, a release at the original site is not possible or if individuals cannot individually be identified after their stay in the shelter [17,18]. It is also known that such release locations are often chosen because of their suitable habitat, while the actual origin of hedgehog individuals is rarely recorded by the animal shelters (in Austria). Therefore, the origin may lie anywhere within a radius of 300 km around the shelter and might as well originate from a different federal province. Moreover, shelters may lack the ability of species identification (*E. europaeus* or *E. roumanicus*) [18]. Despite the proven importance of rehabilitation and translocation as conservation management tools [19,20], the lack of centralized monitoring, regulation, and licensing can lead to uncoordinated translocations, which may have consequences for the wild population at the release site [21].

*E. europaeus* and *E. roumanicus* are not under legal protection by the FFH (Flora-Fauna-Habitat) directive [22] and are not referred to within Austrian hunting regulations [23]. Nature conservation and species protection are regulated on the province level within Austria; therefore, nine different versions of legal regulation apply for the two hedgehog species within Austria. They either categorize hedgehogs as “not protected” or “protected”, with the latter prohibiting the capture, killing, disturbance, ownership, and trade with the corresponding animal. Rehabilitation of wild hedgehog individuals is only allowed under certain circumstances, which include the necessity of rehabilitating injured or underweight individuals as well as the approval of exceptions for the protection of wildlife [18].

Potential outcomes of uncontrolled translocations may be outbreeding depression, accompanied by the loss of local adaptions, or a reduction in genetic variation and changes in the population genetic composition [19,20,24,25]. Even translocations that do not directly lead to gene flow within the affected population may result in genetic consequences through a reduction of the population size, triggered by increased competition or potential transmission of diseases [21]. 

Because human-mediated translocations can also lead to increased incidents of hybridization between species [19,24], a special focus needs to be put on the central European contact zone of *E. europaeus* and *E. roumanicus*, which reaches from Poland and the Czech Republic down through Austria to the border between Italy and Slovenia [9]. It is already known that (un)intentional translocations of various species led to an increase in hybridization and introgression [20]. The degree of hybridization between the two parapatric hedgehog species within their central European contact zone has been indicated to be low so far [8,9,26,27]. Recent investigations relying on a denser sampling of the zone of secondary contact (within Austria), as well as the usage of multiple microsatellite markers, however, indicate that the extent of potential hybridization might be higher than assumed and the actual zone of sympatry and species overlap might be broader [10]. Ongoing work is investigating this further; however, translocations might play a role as an influencing factor for distribution as well as hybridization. 

*E. europaeus* and *E. roumanicus* are known to have overcome the most recent period of repeated glaciations during the Pleistocene era in three different southern refugia. While *E. roumanicus* survived these glaciations in the Balkan peninsula, *E. europaeus* was restricted to the regions of the Iberian and Apennine peninsula [26,28,29,30]. Provoked range shifts, resulting from recurrent restrictions to glacial refugia and expansion during interglacial warmings [28,29,31,32], are reflected in today’s hedgehog genetic structure, as has been shown by various studies [26,30,33,34]. They detected a major genomic division between *E. europaeus* and *E. roumanicus*, and a contact zone separating this divergence between eastern and western Europe [26,30,33,34]. As well as this, a divergence between *E. europaeus* individuals originating from either the Iberian or Apennine peninsula was shown [26,30,33,34]. For *E. roumanicus*, it is suggested that interglacial continental refugia might have led to further genetic differentiation among populations [8]. Nuclear as well as mitochondrial (mt)DNA markers have been used to reconstruct glacial refugia and post-glacial colonization routes of various species worldwide, with European hedgehogs among them [35]. However, it is known that different genetic markers show divergent resolution when observing genetical (sub)structuring and the phylogeographic origin of hedgehogs. While mtDNA markers are proven to be proficient for analyzing the divergence between *E. europaeus*, *E. roumanicus*, and *E. concolor*, as well as intraspecific genetic divergence within *E. europaeus*, nuclear markers were solely able to resolve the major interspecific splits among the three European *Erinaceus* species [26,34]. However, genetical substructuring of existing (sub)species and populations might be affected in regions where individuals of different phylogeographic and genetic origin are intermixed through human-mediated translocations of hedgehogs. In the current study, we aimed to use a set of 55 microsatellite markers, which given their high mutation rate and number should provide a higher discriminatory power than the above mentioned studies. 

Only few studies have focused on the effect of animal translocations on gene flow as a whole [19]. We aimed to generate the basic data necessary to draw solid conclusions on whether the translocation of hedgehog individuals among a certain geographic range would result in the possible disturbance of local genetic structures. Therefore, we raise the question if hedgehog individuals congregated at wildlife shelters display a broader genetical structure and are more diverse than wild hedgehog populations. We answer this question by contrasting different wild hedgehog populations to artificially created “shelter populations”, with regard to their genetic diversity, through Bayesian clustering and the application of principal coordinate analysis (PCoA). The results will be linked with data from questionnaires of Austrian wildlife/animal shelters and shall allow for inferences on the potential impact of hedgehog translocations within the central European contact zone of *E. europaeus* and *E. roumanicus*. Additionally, results will be discussed in a phylogeographic context, as the sampling area is assumed to host both refugial lineages of *E. europaeus*.

## 2. Material and Methods

### 2.1. Sample Collection and DNA Isolation 

A total of 221 samples were used in the present study. Samples were assigned to *E. europaeus* (*n* = 144) and *E. roumanicus* (*n* = 77) by morphological characters. Some samples were already investigated in previous phylogeographical/population genetical assessments [10]. Samples were collected from shelter individuals (*n* = 130) and from wild hedgehogs (*n* = 91). Individuals from one shelter were treated as one population. Corresponding population assignment, as well as the geographical background can be found in Table 1. Figure S1 gives an overview on the sample locations.

Individuals were either sampled through buccal swabs (shelter animals) or muscle tissue (from road fatalities or samples that were taken from museum specimens) [10]. All samples were stored in ethanol until further preparation and analysis. Detailed shelter identity and locations were not given.

DNA isolation followed the methods and procedures described in [10]. For DNA isolation of muscle tissue samples, a small piece of tissue was placed in 500 μL of lysis buffer (2% SDS, 2% PVP–40, 250 mM NaCl, 200 mM Tris-HCl, and 5 mM EDTA, pH8), to which 16.67 μL of proteinase K (10 mg/mL) were added, following a 2.5-h period of incubation at 56 °C. Then, 16.67 μL of RNase (10mg/mL) were subsequently added, followed by an incubation period of 20 min at 37 °C. After the second incubation, 125 μL of 3 M KOAc (pH 4.7) were added and samples were put on ice for 20 min. After a series of centrifugation steps (100 rcf for 1 min, 400 rcf for 1 min, 1700 rcf for 1 min, 7000 rcf for 1 min, and 20,000 rcf for 11 min), 400 μL of the supernatant were mixed with 15 μL of MagSi-DNA beads (size 300 nm, MagSi-DNA beads from MagnaMedics, Geleen, The Netherlands), as well as 600 μL of binding buffer (2 M GuHCl in 95% ethanol) and incubated at room temperature (5 min). To separate the resulting supernatant from the beads, the samples were placed on the magnetic separator SL-MagSep 96 (Steinbrenner, Germany) for 1 min. Two washing steps with 80% ethanol (600 μL each) followed. After discarding the supernatants, magnetic beads were air-dried at room temperature for 10 min. Two elutions were acquired, with 30 and 50 μL of preheated (65 °C) elution buffer (10 mM TrisHCl, pH 8), mixed with the beads, and incubated for 5 min at room temperature. DNA isolation of buccal swab samples followed the same procedure, with the exception that the product of lysis was filtered through NucleoSpin filter columns and centrifuged for 1 min at 562× *g*. Buccal swab samples were eluted in 20 and 25 µL of elution buffer [10].

### 2.2. Molecular Marker Enrichment and Amplification

A short sequence repeats genotyping by sequence approach (SSR-GBS), developed by [10], was used within the present study. Amplification of the microsatellite regions within the genomes of *E. europaeus* and *E. roumanicus* was conducted through multiplex PCR. In total, 55 different primers were available for this approach, 25 and 30 primer pairs for the species *E. europaeus* and *E. roumanicus*, respectively. A list of all primers that have been used for multiplex PCR can be found in the Supplementary Materials (Table S1). These primer sets were developed and improved by [10]. Amplification primers are built up by specific sequences that are elongated by Illumina P5/P7 sequences, which correspond to the Illumina adapter in the sequencing process. The forward primers were elongated with a part of the P5 motif (TCTTTCCCTACACGACGCTCTTCCGATCT) and the reverse primers with a part of the P7 motif (CTGGAGTTCAGACGTGTGCTCTTCCGATCT). The Multiplex PCR Kit from QIAGEN was used for the performance of multiplex PCR. Therefore, 1 μL of template was added to a prepared mix of 5 μL of QIAGEN Multiplex PCR Master Mix (Qiagen, CA, USA), 3.5 μL of H20, and 0.5 μL of a specific primer mix (1 μM). PCR was conducted using the following temperature profile: 95 °C for 15 min; 30 cycles of 95 °C for 30 s, 55 °C for 1 min, and 72 °C for 1 min; with a final extension at 72 °C for 10 min. For the evaluation of successful amplification and quality of the PCR products, agarose gel electrophoresis was conducted to visualize PCR results. 

Purification of PCR products in preparation of index PCR was conducted using an inverse magnetic separator. A total 6-μL sample volume was mixed with 4.3 μL of magnetic beads and incubated at room temperature for 5 min. The DNA, bound to magnetic beads, was washed twice with 200 μL of 80% ethanol for 45 s. After discarding the ethanol, the magnetic particles were air-dried for 5 min and DNA was eluted with 17 μL of preheated (65 °C) elution buffer (10 mM TrisHCl, pH 8).

Index PCR was used to allow for the individual identification of the pooled samples with specific forward and revers primers through specific assignment of indices. On the one hand, the used primers help binding to the before amplified P5/P7 part of the primers used in multiplex PCR. On the other hand, they allow for binding to the flow cell in Illumina Sequencing and label the sample with a unique eight-bp index information, which helps to assign sequenced genotypes to single samples (P5: AATGATACGGCGACCACCGAGATCTACAC [Index] ACACTCTTTCCCTACACGACG; and P7: CAAGCAGAAGACGGCATACGAGAT [Index] GTGACTGGAGTTCAGACGTGT). For index PCR, 1 μL of clean PCR product was mixed with 5 μL of Multimix (QIAGEN), 2 μL of specific P5 forward primer, and 2 μL of specific P7 reverse primer. The following temperature profile was used for the performance of index PCR: 95 °C for 15 min; 10 cycles of 95 °C for 30 s, 58 °C for 1 min, and 72 °C for 1 min; with a final extension at 72 °C for 5 min [10].

### 2.3. Illumina Sequencing and Sequence Data Analysis

Following index PCR, samples were pooled and sent to the Biozentrum LMU in Munich, Germany, for sequencing in an Illumina MiSeq machine in both directions/through paired-end sequencing in a sequencing by synthesis approach.

The short sequence repeats genotyping by amplicon sequence (SSR-GBAS) method used in this paper follows the pipeline described in [10,36], available at [37]. Amplicon sequences are being used for the determination of genotypes. In this context, alleles are defined according to their length and the occurrence of single nucleotide polymorphisms (SNPs).

Sequences resulting from the Illumina run were supplied in two FASTQ files (read 1 and read 2), containing all sequences per index. The following processing and treatment of the samples was based on a combination of custom-made scripts, as well as third party programs [10]. This included the quality control of single bases, as well as each read, followed by the trimming of low-quality regions (Phred < 20, according to [10]). Sequences were aligned using the program PEAR [38], meaning that paired reads (read 1 and read 2) were merged. The necessary overlapping range was set to a minimum of 10 bp, with “a *p*-value below 0.01 for the highest observed expected alignment scores” [10,38]. Markers were explicitly designed to generate overlapping fragments of approximately 300 bp. Overlapping sequences below 250 bp and non-overlapping reads were not considered during further steps. Through a “demultiplexing” step, it was possible to identify primer sequences on each side of the merged reads and separate them per locus using the script primer_demultiplex.py [10,37], as merged reads were supposed to begin with the forward primer and end with the reverse primer sequence, based on library preparation. Finally, sequences were sorted by locus and sample, resulting in corresponding files, which were used for further genotyping analysis [10].

Allele definition was largely based on two major steps: (1) Sequence length, as well as (2) the possible occurrence of SNPs in each separate length class. Within each sample, each marker was examined for its most frequent sequence length class. This was done through a custom-made script (Rscript_Markerlength_develop_Color.R, [10,37]) and manually controlled based on length histograms. Loci were considered to be homozygous if they comprised a certain length with a frequency equal to or above 90% among all reads for the respective marker. If a locus showed two lengths with a frequency greater than 90% of all reads (and those frequencies differed by less than 20%), the genotype was considered heterozygous. As well as the calling for alleles based on length frequencies, the employed script (Rscript_Markerlength_develop_Color.R) checked for possible stutter within the selected alleles [10,37]. The remaining steps were preformed using the script Sequence_Allele_Call.py [37]. The various reads within the most frequent length class(es) of a (homozygous) locus were merged into one consensus sequence. Therefore, nucleotide positions were considered to be homozygous if they showed a frequency above 70% for a single position, and to be heterozygous if the frequency of a nucleotide within a single position was below 70%. Loci, within a specific sample, that had already been defined as homozygous based on their sequence length class could be considered heterozygous based on the two most frequent nucleotides for a position. Nucleotide positions were considered to be linked if more than one potential SNP (single nucleotid polymorphism) occurred in a sequence. For samples already defined as heterozygous based on length class, it was decided to choose the most frequent SNP combination. Based on the called alleles from all samples, a codominant matrix was set up, as input for subsequent population genetic analyses within different standard programs [10]. This matrix consists of two specific numbers, corresponding to unique sequences (i.e., specific alleles), for each investigated locus, of every sample.

### 2.4. Population Genetic Analysis

After the initial sequence analysis and exclusion of markers with too much missing data, 41 microsatellite markers were valid to be used (see Figure S1) for population genetic analysis of the 221 hedgehog samples. 

Specific analyses were conducted in the following different approaches: (1) Analysis of all 221 hedgehog samples; (2) intraspecific analysis for the two respective species *E. europaeus* and *E. roumanicus*; as well as (3) a separate analysis of shelter and wild populations (on an inter- as well as intraspecific level).

The program STRUCTURE v.2.3.4 [39] was used for the detection of the underlying population structure within the investigated set of hedgehog individuals, based on Bayesian clustering (Markov Chain Monte Carlo, MCMC) of multilocus genotype data [39]. For all conducted approaches, the “Length of Burnin Period”, as well as the “Number of MCMC Reps after Burnin” were selected as 10.000. An admixture model, with correlated allele frequencies was chosen for calculation. Calculations were conducted with 5 iterations. 

To choose the most likely value for all observed Ks and thereby determine the most likely number of populations (genetic groups) to be found within the underlying set of samples, the “STRUCTURE HARVESTER” [40,41] was used. To summaries all iterations of each K into one single summary output and graphically represent the results calculated within STRUCTURE v.2.3.4, the web portal CLUMPAK (Cluster Markov Packager Across K) was used [42,43].

The Excel Add-In GenAlEx (Genetic Analysis in Excel) [44,45] was used for principal coordinate analysis (PCoA) using the covariance-standardized setting, and calculation of the standard population genetic parameters, like the observed and expected heterozygosity (He, Ho), fixation index (F), percentage of polymorphic loci, and number of alleles (Na).

## 3. Results

**Figure 1:**
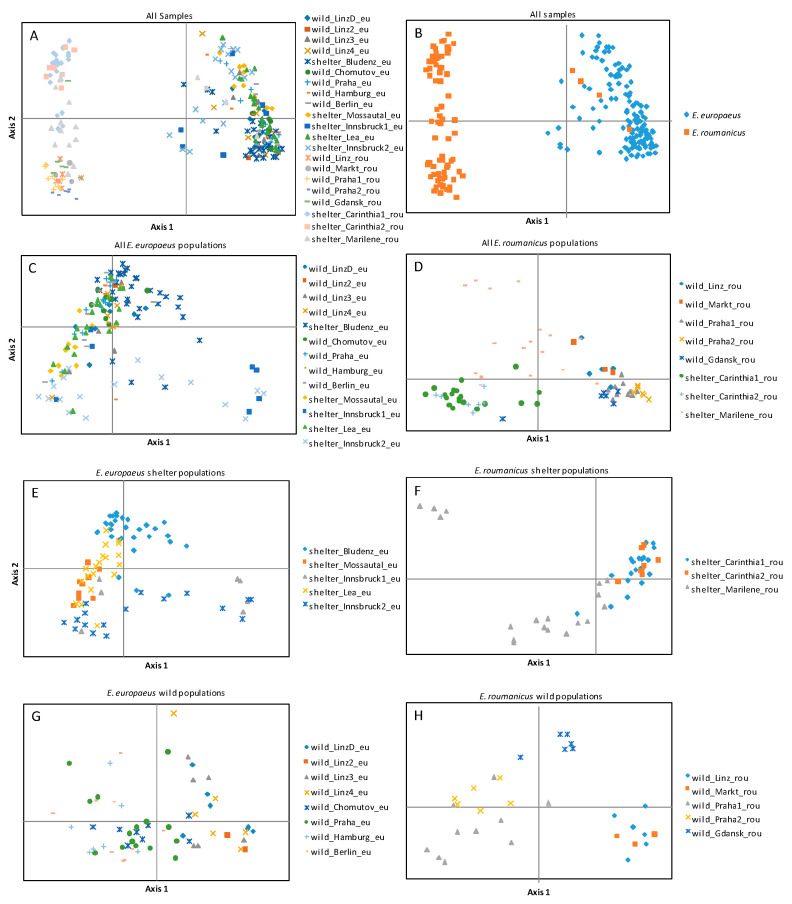
Principal coordinate analysis from genetic distances of the whole dataset ((**A**) for Populations, (**B**) for species), All populations divided per species (**C**,**D**), shelter populations (**E**,**F**), and wild populations (**G**,**H**). C through H are divided in *E. europaeus* (left) and *E. roumanicus* (right).

**Figure 2:**
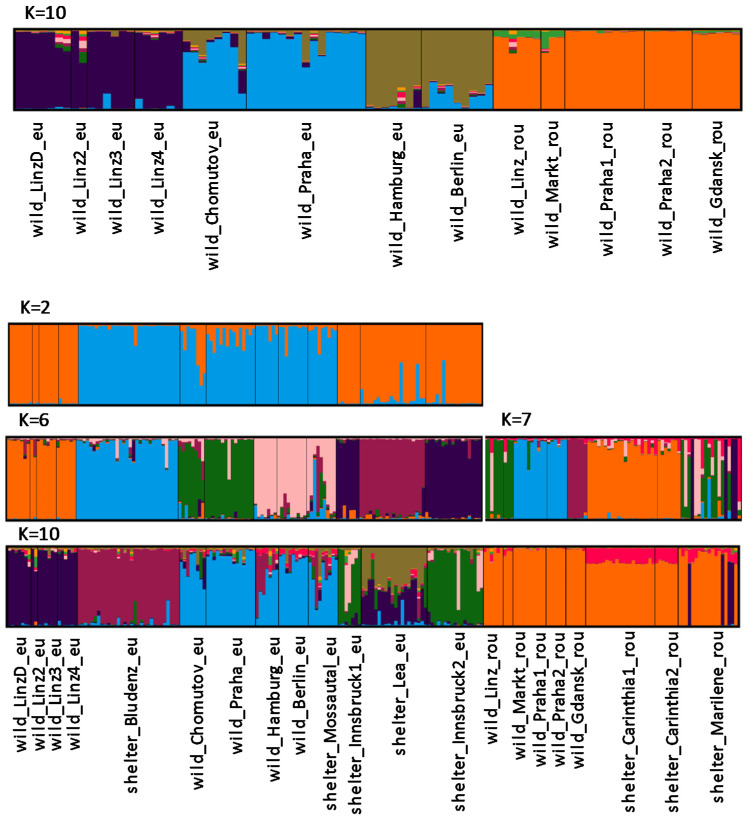
Structure analysis for the datasets of all wild (upper panel), all *E. europaeus* (K = 2, upper middle panel and K = 6 lower middle panel), all *E. roumanicus* (K = 7 lower middle panel, right), and all populations (K = 10 lower panel). The shown K corresponds to delta K_max_ (*E. europaeus* K = 2 and *E. roumanicus* K = 7) or suboptimal delta K (all populations K = 10; all wild populations K = 10, *E. europaeus* K = 6).

**Figure 3:**
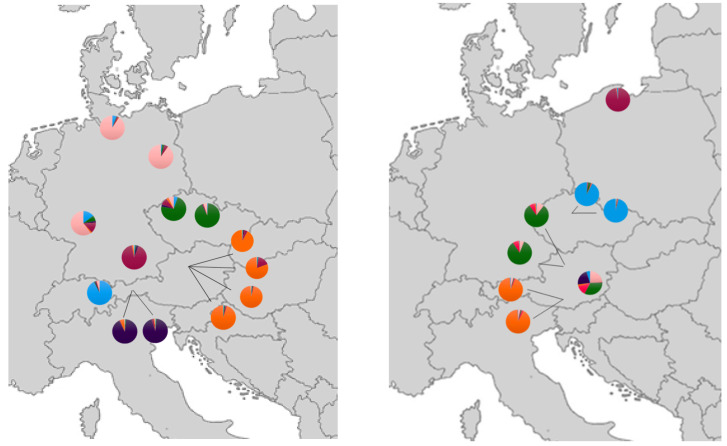
Spatial patterns of the genetic structure within *E. europaeus* (left panel) and *E. roumanicus* (right panel) shelter and wild populations. Raw data used and color-coding refer to structure analysis within the single species like shown in Figure 2 (K = 6 for *E. europaeus* and K = 7 for *E. roumanicus*). Basic map was taken from [46]. Localities are listed in Supplementary Materials Figure S1.

The data included in this study were obtained from different Illumina runs. Files corresponding to one sample containing all markers were used as input for quality control, merging of reads, and the allele call pipeline. After the exclusion of markers with more than 50% missing data, the matrix contained 41 primers for 221 samples. Primer characteristics were largely congruent with our earlier analysis [10] and are therefore not reported here. As expected, strong differentiation between *E. roumanicus* and *E. europaeus* was obvious, and intermediate specimens between the species recognized previously were not included in this study. No early generation hybrid was detected in this dataset and no individuals were positioned between the main clusters characterizing the two species (Figure 1). Within the single species, moderate genetic structure was indicated. In the analysis of all wild populations (K = 10), three clusters in *E. europaeus* (one for the populations in Linz, one for the Czech Republic (Prague and Chomutov), and one for the northern German populations of Berlin and Hamburg) are opposed to one cluster for all *E. roumanicus* populations (Figure 2). When looking only at clusters within one species, *E. roumanicus* was divided into three groups, one for Linz and Markt, the second for the populations in Prague, and the third for the population in Gdansk (Figure 2). When the populations from shelters are included, subdivision becomes clearer, as some of the shelters are from areas where no wild population was collected (Figure 2, Figure 3). For *E. europaeus*, despite the optimal K according to the Evanno method being 2, at K = 6, a second optimum is indicated and three clusters of the wild populations, as well as the shelter from Bludenz (Vorarlberg, western Austria), Innsbruck (Tyrol, western Austria), and Bavaria (shelter “Lea”; southern Germany) form their own clusters. The shelter from Mossautal was assigned to the same cluster as the northern German populations. In *E. roumanicus*, the analysis of all samples results in one additional cluster for the shelters in Carinthia and the shelter in Graz (shelter “Marilene”), which appears very admixed. In the shelter of Graz, we have some individuals that cluster together with *E. europaeus* (Figure 1), indicating that this population is not homogeneous. 

**Figure 4:**
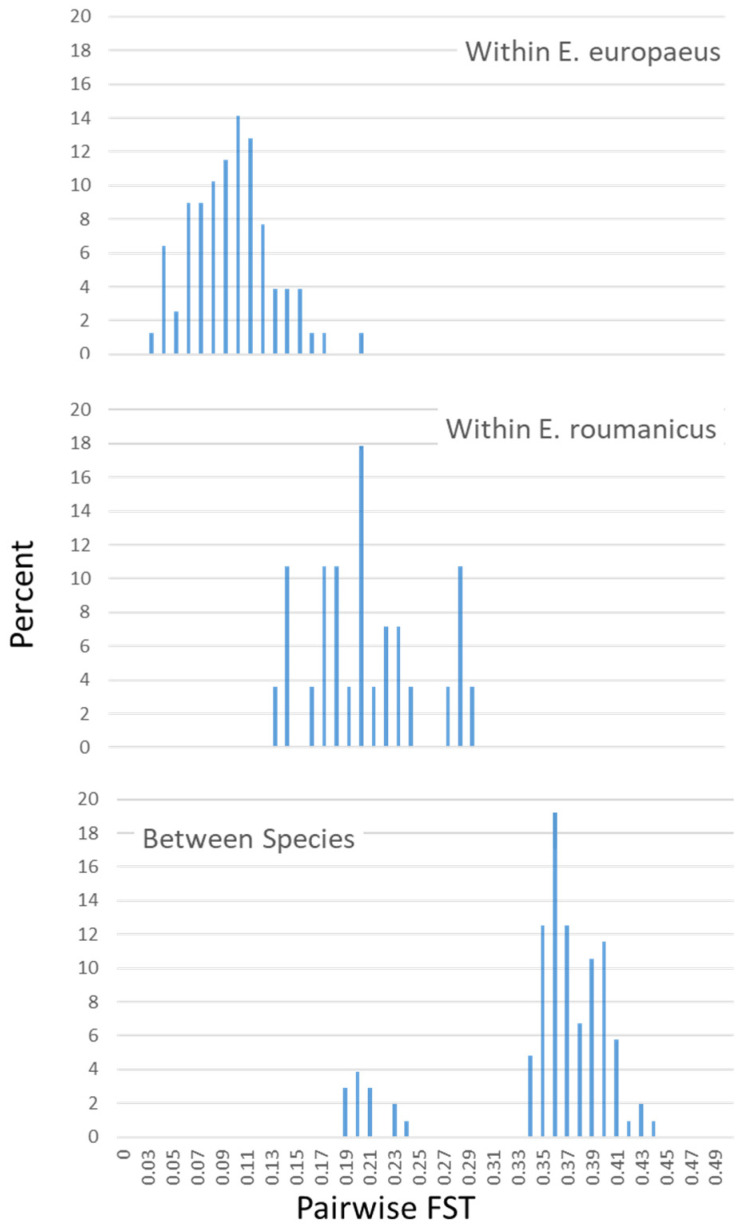
Frequency distribution of pairwise F_st_ between populations of *E. europaeus*, *E. roumanicus*, and between populations of both species.

Genetic differentiation between populations seems to be rather high with the marker system used. While the pairwise F_st_ between populations of the two species ranges from 0.3 and 0.4, intra-species values are between 0.02 and 0.21 in *E. europaeus* and between 0.13 and 0.3 in *E. roumanicus* (Figure 4). Low inter-species values (between 0.19 and 0.24) are attributed to the differences of admixed shelter populations where both species were included. 

## 4. Discussion

In concordance with previous studies on hedgehog genetic divergence and phylogeography [9,10,26,30], the results support a clear delimitation of the two species *E. europaeus* and *E. roumanicus*. The data allowed for phylogeographic inferences on factors shaping the genetic diversity of hedgehog populations. 

Bayesian clustering revealed a most likely K = 2 for *E. europaeus* individuals within our dataset. This is corresponding to existing phylogeographic lineages of *E. europaeus* [26,30], based on analysis of mitochondrial markers. Hedgehogs survived the last glacial maximum in Mediterranean refugia and later colonized Europe northward in connection to spreading forests [28]. One of the lineages has Iberian origin [28,29] and recently is also occupying France, England, Switzerland, and southwest Germany [26] For the first time, we might have detected this lineage in Austria, which could be attributed to sampling gaps in previous studies. Due to its proximity to Switzerland and Germany, one could propose a possible existence of this lineage within Austria´s most western range. This is furthermore strengthened by the congruent assignment of the Bludenz and German (Berlin, Hamburg, Mossautal) populations within STRUCTURE analysis (K = 2), despite their relative geographic distance and the genetic distinctiveness of the Innsbruck population, despite its relative geographic proximity. Investigation on mitochondrial markers to verify the origin of this lineage is currently being undertaken. 

The Apennine lineage of *E. europaeus* expanded out of the Italian Peninsula northwards through Austria, Switzerland, Germany, the Netherlands, Scandinavia, and Estonia [26]. In contrast to many other European species, hedgehogs have been shown to overcome the mountain barrier of the Alps and colonize Europe out from Italy [28,29]. The distinct pattern of the Bludenz population indicates the possibility that lineages of *E. europaeus* originating from the Iberian and Apennine Pleistocene refugia both occur in Austria. The separation of Linz and also Czech samples of K = 3 might be explained on their proximity to the zone of secondary contact and underlying genetic divergence or introgression [8]. Their genetic set-up might be established on possible introgression with *E. roumanicus* individuals, as they are situated in the potential hybridization zone. A possible influence of individuals from the secondary contact zone on the analysis of genetic divergence within the species has already been indicated by [8]. They also concluded that populations from the contact zone are different from the remaining study area, which they attributed to processes acting at parapatric range edges. 

*E. roumanicus* showed a more pronounced genetic structure when looked at pairwise F_st_ than *E. europaeus*. It is suggested that only one lineage of *E. roumanicus* emerged from the southern refugium of the Balkan Peninsula [26,29,30]. Intraspecific structure analysis of *E. roumanicus* indicated a differentiation of all populations at K = 7 and populations of Linz, Prague, Gdansk, and Carinthia clearly differentiated from each other and stayed in a uniform pattern corresponding to their geographic origin. This pattern is in contradiction to previous shallow population structure found in this species [8,9]. It may be explained as a result of the hybrid zone dynamic with two populations (Linz and Praha) within the zone of sympatry [8]. Populations found within the cities of Linz (federal province of Upper Austria) and Prague are comprising both species. Individuals that were indicated as hybrids in the previous study with the same marker set [10] were not part of the populations included in this study. The division between the species is supported by a high F_st_; however, also within one species populations were divided by F_st_, indicating a moderate to high level of genetic structure. Moreover, their intraspecific F_st_ distribution is multimodal with some comparisons on the same range of the interspecific F_st_ values. Neutral markers exchanged between the two species could increase the signature of genetic differentiation between populations close and distant to the contact zone. For this study, only two areas with sympatric populations could be included so no specific analysis on introgression was performed. This might apply to *E. roumanicus* to a higher extent than to *E. europaeus* given their F_st_ distribution patterns. Ongoing work will investigate this further. 

Landscapes across Europe appear to be highly fragmented due to anthropogenic infrastructure and various forms of human land use [4,12,13]. Hedgehog populations subjected to restricted gene flow due to other factors than geographic distance alone have been found in the UK [7] and in Switzerland [14]. Especially within areas where the hedgehog´s distribution range is structured by major geographic barriers, like rivers or mountain ranges (as seen in the shelter populations of Innsbruck), these impediments might be circumvented through human-mediated infrastructure (such as bridges) or translocations. This artificial enhancement of species distribution may lead to a promotion of gene flow in specific areas. Whether or not this enhancement in gene flow has positive (enrichment of locally isolated populations in fragmented environment through an increase of genetic variation, [19]), negative (disturbance of locally adapted populations and outbreeding depression, [25]), or neutral effects on natural wild hedgehog populations remains the subject of future investigations. 

The shelter populations observed within this study did not generally comprise a higher genetic diversity as could be expected because they comprise a mixture of individuals from a wide geographical area. While the shelter populations of Carinthia, Bludenz, and Bavaria appeared homogenous, the shelter population of Graz was highly diverse, with the appearance of both species. Personal information given in an interview of the shelter indicated that no differentiation of the two species is considered during sheltering. The two shelter populations from Innsbruck show a certain level of subdivision into two subpopulations in the PCoA. The exact origin of these samples is unknown; however, it is likely that these individuals could stem from both sides of the Inn, a river running through the federal province of Tyrol in the direction of south-west to north-east, which could constitute as a natural barrier [14]. 

The *E. europaeus* and *E. roumanicus* distribution within their central European contact zone is already known to be syntopic [9]. While increased landscape fragmentation might promote the limitation of gene flow [4,13,14] as well as a varying abundance and density of both hedgehog species in different regions [9], translocations have the possibility to interfere with these structures. Moreover, translocations might broaden the zone of overlap and might enhance the potential for hybridization. Using questionnaires, it was assessed that most shelters can differentiate between the species but regard species delimitation as irrelevant in practice [18]. The discovery of both species, *E. europaeus* and *E. roumanicus*, within a single shelter population (shelter Graz) shows shelters potential to interfere with the two species distribution range. In the thesis of [18], 8 out of 12 wildlife shelters (corresponding to 6 out of the 9 federal provinces) in Austria hosted both species. This is reinforced by shelter habits in animal translocations. Since wildlife shelters hold most of their hedgehogs in groups, the exact identification of single individuals is often neglected. The majority of wildlife shelters in Austria give rehabilitated hedgehogs back to the people that brought them (11 out of 13 shelters; [18]); however the individual that is handed over might be confused. A release at the place of origin is often not possible (due to a lack in individual identification; an unknown place of origin; or the place of origin being considered inappropriate; 8 out of 13 shelters in [18]), wherefore 6 out of 13 shelters turn to people voluntarily offering releases at places considered suitable [18]. Eventually, distances between the place of origin and release site are known to vary from 5–300 km in different shelters [18].

Studies mostly discuss the effect of translocation as the effect on the survival of individuals with lower fitness, which could interfere with natural selection [25]. Potential results might be relaxed selection pressure on survival during winter and a reduction of the adaptive response. In addition, more virulent forms of parasites and diseases could be kept in populations, allowing more damaging forms of diseases to develop [25]. This means that even releases that do not directly result in gene flow can have genetic consequences if they reduce the local population size by disease transmission or increased competition [21]. Various studies showed the capability of rehabilitated juvenile and adult hedgehogs to survive after their release [47,48]. Additionally, an improvement in the fitness of hedgehogs in Great Britain following temporary captivity was found [17]. The same study showed that rehabilitated and in turn released hedgehogs did not affect wild individuals through an increase in competition. This was attributed to suburban gardens serving as highly productive feeding sites [49]. 

Human-caused augmentation of hybridization pressure due to the translocation of individuals beyond the species range had been considered [19]. For the two parapatric hedgehog species *E. europaeus* and *E. roumanicus*, hybridization is recognized, but the degree seems to be very low in terms of early generation hybrids [9,36,50]. Long-term patterns of continuous introgression and its effect on genetic structure, like outlined above, could, however, very well be impactful. In the future, the data and marker system presented here, allowing for easy reproduction of genotyping data, will allow to monitor and investigate hybridization likelihood in detail. 

Only few studies or data can be found about how translocating animals from wildlife shelters affects gene flow [19]. In our study, we found indications that genetic structure formed by landscape elements could be impacted over longer time scales when individuals from both populations are included in shelters. The example of the shelters from Innsbruck shows that this is possible. Even though, with a few exceptions, we did not find genetically strongly deviating individuals in the shelter populations, personal observation shows that hedgehogs can be transported via several hundred km for care and subsequent release. Even if this is unlikely to have a pronounced effect on the population structure [51], the consideration of the source for release is important, especially if the region is subdivided by migration barriers for the species. 

## 5. Conclusions

Hosting hedgehog individuals in shelters can promote translocation of individuals because it can bring together animals that are isolated by dispersal barriers. In the zone of sympatry, the two different hedgehog species *E. europaeus* and *E. roumanicus* are sheltered together and are not always differentiated. Our data show that such cases occur, even if in most shelters a genetic effect is not seen. Shelter individuals should therefore be released under the consideration of natural barriers and treated as a species that can express small-scale genetic structure. Future research will investigate this further. Because the main consideration of shelters is animal welfare, also individuals from distant locations can be taken in, but for the individuals tested, this did not apply. 

## Figures and Tables

**Table 1:** Sample information and populations. The table gives an overview on the used population name, number of individuals within each population, species assignment, and geographical origin. Abbreviations given: AUT = Austria, CZE = Czech Republic, GER = Germany, POL = Poland.

Population	# Individuals	Species	Geographical Origin
wild_LinzD_eu	7	*E. europaeus*	Linz/AUT (wild)
wild_Linz2_eu	2	*E. europaeus*	Linz/AUT (wild)
wild_Linz3_eu	6	*E. europaeus*	Linz/AUT (wild)
wild_Linz4_eu	6	*E. europaeus*	Linz/AUT (wild)
shelter_Bludenz_eu	31	*E. europaeus*	Bludenz/AUT (shelter)
wild_Chomutov_eu	8	*E. europaeus*	Chomutov/CZE (wild)
wild_Praha_eu	15	*E. europaeus*	Prague/CZE (wild)
wild_Hamburg_eu	7	*E. europaeus*	Hamburg/GER (wild)
wild_Berlin_eu	9	*E. europaeus*	Berlin/GER (wild)
shelter_Mossautal_eu	9	*E. europaeus*	Mossautal/GER (shelter)
shelter_Innsbruck1_eu	7	*E. europaeus*	Innsbruck/AUT (shelter)
shelter_Lea_eu	20	*E. europaeus*	Bavaria/GER (shelter)
shelter_Innsbruck2_eu	17	*E. europaeus*	Innsbruck/AUT (shelter)
wild_Linz_rou	6	*E. roumanicus*	Linz/AUT (wild)
wild_Markt_rou	3	*E. roumanicus*	Linz/AUT (wild
wild_Praha1_rou	10	*E. roumanicus*	Prague/CZE (wild)
wild_Praha2_rou	6	*E. roumanicus*	Prague/CZE (wild)
wild_Gdansk_rou	6	*E. roumanicus*	Gdansk/POL (wild)
shelter_Carinthia1_rou	21	*E. roumanicus*	Carinthia/AUT (shelter)
shelter_Carinthia2_rou	7	*E. roumanicus*	Carinthia/AUT (shelter)
shelter_Marilene_rou	18	*E. roumanicus*	Graz/AUT (shelter)

## Supplementary Materials

The following are available online at https://www.mdpi.com/2076-2615/10/9/1452/s1. Figure S1: Map with locations of populations included in the study; Table S1: Primers used in the Multiplex PCR approach for sequence analysis of *E. europaeus* and *E. roumanicus*.
