# Supplementary material for: Evaluating the Impact of Wildlife Shelter Management on the Genetic Diversity of Erinaceus europaeus and E. roumanicus in Their Contact Zone
Source: Animals (Basel). 2020 Aug 20;10(9):1452. doi: 10.3390/ani10091452 (PMC7552763; doi:10.3390/ani10091452)
Supplement: Supplementary file 1 [file animals-10-01452-s001.pdf]

animals MDPI

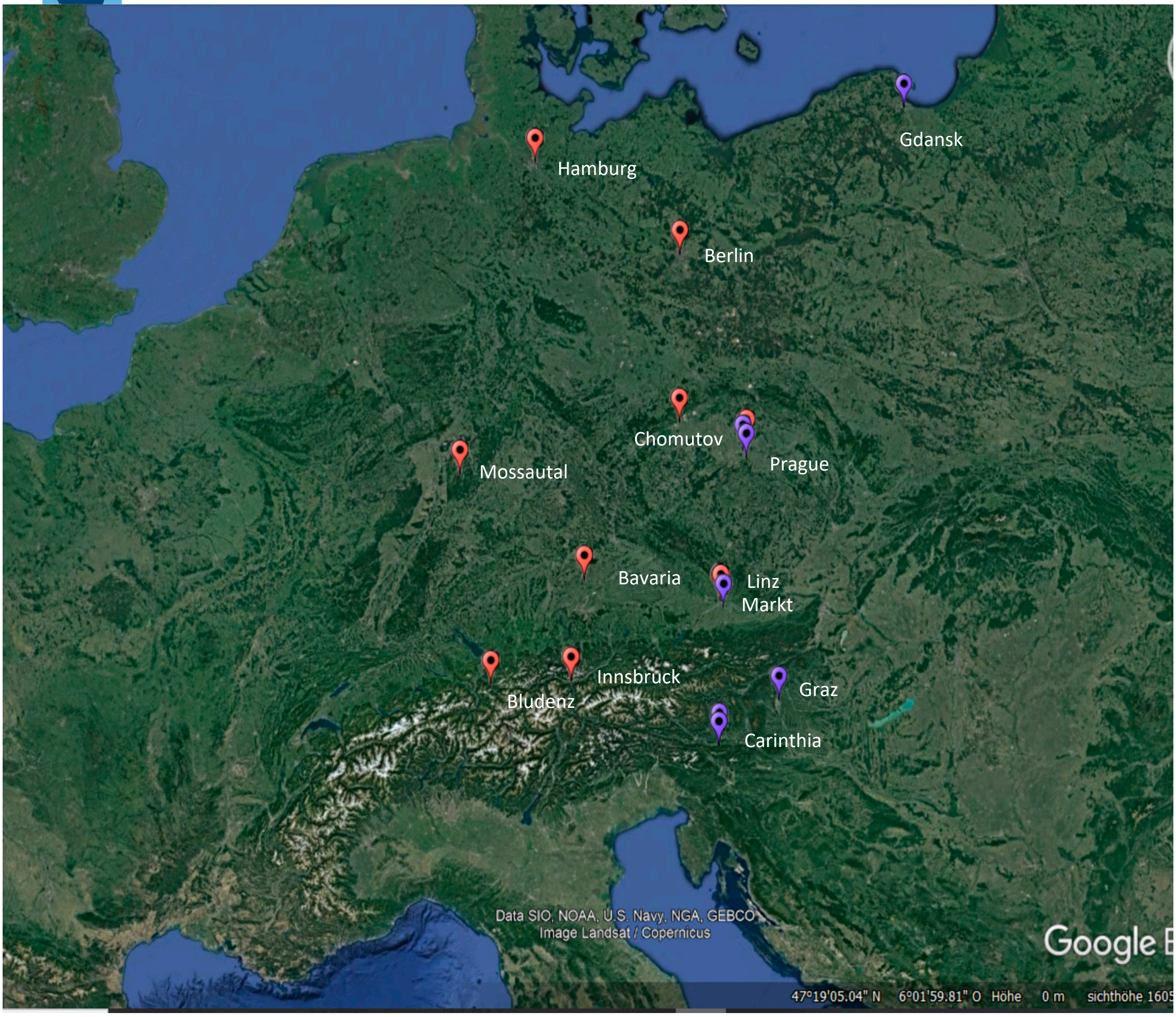

Figure S1. Map with locations of populations included in the study. Bludenz, Innsbruck (two populations, Carinthia (two populations), Graz, Bavaria and Mossautal are shelter populations. Red and blue pins refer to populations of *E. europaeus* and *E. roumanicus*, respectively.

Table S1. Primers used in the Multiplex PCR approach for sequence analysis of *E. europaeus* and *E. roumanicus.* The table shows the primer name – including the repetition motif, the number of repeats of the motif in the original sequence, the primer sequence (forward and reverse), the allele length variation (Amplicon length variation) and the corresponding primer mix. Primer mixes HH1, HH2 and HH3 are supposed to amplify loci within *E. roumanicus*, whereas primer mix WHH1 contains primers that were designed from *E. europaeus*. The missing data column indicates the amount of missing data over all loci after initial sequence analysis. Primers that are mentioned underneath the boundary line did not go into further population genetic analysis as they showed over 50% missing data among all analysed loci (primer development by [10]).

Primername	Nr. Repeats	Forward	Reverse	Amplicon length variation	Primermix	missing data [%]
HH1_AC	16	TCATGCTAGGCACTGCTATT	AAGTGCAATCAGACCAGTGA	454-486	HH1	27,15
HH11_AAAG	7	ACGTTCCTCTCTGGGGAATA	TTCAAGACCCTGTTCTCCAC	428-460	HH1	6,33
HH13_AAGG	11	AGGTAGAAGGCAGACAATGG	TTGAAACACTGACGTAGGCT	428-476	HH3	1,36
HH16_AAGAA	12	CACTAGGCAGAAAACACACG	ACCACAGATGCTGTAGACAG	425-480	HH2	1,81
HH18_ATTTT	9	TAGCCTGGGGGAAAATCAAG	GCAATTTCCAGTAGAGGGGA	438-475	HH1	4,98
HH19_GAAAT	12	CCTTGCTTGTTTCCTAAGCC	ATCACTGGGACTCCCTCAAT	472-527	HH3	3,17
HH2_AC	16	TGGGTAGCAGCTAAAGGAAG	GACAAAATCCTCCCTGGCTA	448-482	HH2	8,60
HH20_TATTC	11	TGGATGGATGGAAAACTGAGA	GGGTGCTGATTCATCTACCT	477-522	HH3	28,05
HH22_AG	15	ACGGAAGGAAATACTGCCAA	CCTTCCCCTTTGTGAGAACT	470-502	HH3	0,90
HH24_GCA	7	TTCTGAGGTCTCATCTGTGC	CTGTCTGTGGTCCAGGAAAG	452-452	HH3	1,36
HH26_CAA	10	TTAAGGAACTCAGGGTTGGG	GTGTCAATGGAAGCAAAGCT	487-502	HH1	17,65
HH27_TTG	9	AGATGCTCAAGGGAAACTGA	TCACAGCATACTTAGGAGCC	452-477	HH2	0,90
HH28_TTG	8	CCTAGTGGTAGCTTCTCACA	TTGGCTCCAGTCAAGTTCTC	440-452	HH3	4,52
HH29_TCAA	7	CTTGTGCACTGTGATGTGAG	ACGAAGTTTCCAGGAAGCTC	486-494	HH1	19,00
HH3_CA	17	GGCAGACTGTCTAGTTCACA	GGTCTAGGACTGCACCATTT	476-512	HH3	38,01
HH30_TCAC	8	AGCGTTAAATACATCCGGCT	AACCCATTGACTCTCTGACA	430-458	HH2	0,00
HH31_AACA	7	GGAAGCGCCTTCATTATAGC	CTCCTGTCACTAGCCAGAAG	476-484	HH1	5,43
HH34_TCTA	10	AGACCACAGTGTCCAAGTTT	GATTTCCCCTGTGTAGGTGA	428-466	HH2	1,36
HH35_AAAAT	6	TGGGTTGTAGATAACACTCA	ACTGCAGGTGGAGATATGTG	464-482	HH2	28,05
HH36_GAAAG	9	ACAGTGAAGACAGGGAAGC	CTTAAAATGGCTAAGGTGGT	452-517	HH1	5,43
HH37_CTTTT	7	CTGCAGTTTGCTCTGGATTC	AAGAAAGAAGCCCTTGTCCA	446-480	HH2	2,26
HH4_CT	13	TCAAGGAGTGTGTTGACCAG	ATCCCTTTGCTCAGCCAAT	452-462	HH1	8,14
HH5_GA	17	TTTCTTGCTCAGAACCCTGA	CAGGGGGAATGCTTTTCAAG	446-480	HH2	32,58
HH8_ATT	8	CCTCCAGGAGAGATTTTGCT	CAAATGAGTGGAAGCCATGC	489-498	HH3	25,79

HH9_ATT	13	GTTGACACTCTTTGCTGCTT	CAAGTCCTCACTAAGCCTGT	425-444	HH1	20,81
WHH10_AAAAC	7	ATAGCTGGATAGTGGTCTGG	ACATCTTTTCTTCCTCACAGT	398-433	WHH1	1,81
WHH11_CTTC	10	AGTCACCATTCTCCACTTTC	ACCCTGAGTGAAGAAGGATA	413-435	WHH1	45,70
WHH12_GAAA	8	AACTCAAATTACAAGGGGCC	TCCAATAACTAGGGGTTTAAGT	386-474	WHH1	6,79
WHH14_ATAG	10	AAAAGGACCTAAATGGGAGG	ACAGGGAACAAAGATGCTTA	376-408	WHH1	2,71
WHH16_TTAA	7	GTGTAAAGCAGTATGTTGCC	AATACAGTGTACAAGGACGC	407-419	WHH1	28,51
WHH19_TTCT	13	AGAGATCAGACTAACGTTTTT	GGGGAGAATTTGGTACTGTA	402-443	WHH1	37,56
WHH21_TTTA	7	ACTTCACTATCACCCTTCAA	ACTTGATTTGTTTATGGGGTG	395-403	WHH1	53,85
WHH23_TGGA	13	TCTTCCCTTAAGCTACTGGA	TCTCAATTGTTTAGACATTGAGT	386-414	WHH1	8,60
WHH29_CT	15	CATTACCGTGCACACAGA	GTTTGATCCCCACCACTTAA	406-422	WHH1	26,70
WHH30_CT	17	TCTCATTGGATAGTGCACTG	TGCCTAATAGCAAATACACA	405-441	WHH1	30,32
WHH32_GT	13	CAGTCAATGCATTCCCAATC	TGTGTGGTACAGGGAATAGA	415-451	WHH1	47,51
WHH33_CA	11	AGAAAAGACCTCAGGAGACT	CCTGGAGAGTGGAAAAGTTA	424-456	WHH1	51,58
WHH5_AAAAT	8	CACCAGGTTAAGCGTACATA	AAAAGTGCTACTAGGGAAGC	NA	WHH1	38,91
WHH7_TCTTT	9	TTAGCTTGGTTTTCACAGGT	GAGTGGCAGTCTTCAAGTAG	384-419	WHH1	7,69
WHH8_TTCCT	10	ATAGGAGGACTGGCGATC	AATGGAGGGAGTAGATGGG	364-424	WHH1	1,36
WHH9_TTTCT	10	TTCAATCTCAAGTACCACATT	GATGCACCTGGTTGAGAG	384-414	WHH1	45,25
HH10_AAAG	11	AAGCACAACAACAATGGCAA	ACGTACTGAGCCTTTCAAGA	437-545	HH2	69,11
HH25_TAC	9	TGTTATCATGCCTGAGGACC	CTGGTTGGGAAGAGAAACCT	NA	HH1	94,60
HH32_ATCT	7	TGACAGTGTGTGGTTGACTT	TTCACCATCGCAGAGAACAT	NA	HH3	98,06
HH6_AAT	16	CTCTTGGTGTGCATGACAAG	CTGTGACCCGTGTAGTTGG	NA	HH1	97,62
HH7_AAT	10	ACCATAGCTTTGTAATCTCCT	AGGATGATGGCCCTTTGAAA	445-463	HH2	65,44
WHH1_AAAAT	7	GGGTAAAACAGGTCTGATGT	AAACTTGTCAGGAAGCAGTT	382-407	WHH1	64,15
WHH13_TTTA	7	TTTCACTCTGGGTTACTGTG	AAGTGGTGCAACTCTAAGAC	386-395	WHH1	88,77
WHH15_ATAA	8	ATACTCCCAGCCTGTTTCTA	ACCTCCCAAGAACTCTATCA	367-390	WHH1	76,67
WHH18_AATA	8	ACTCAAAAGTTTTCCACCCT	TTTTAGGCTCTGCTCTTCTG	403-411	WHH1	77,97
WHH20_TAGA	8	TGCACATTACAATGTTCAAGG	TACATCAGGGAGAGTACAGG	NA	WHH1	100,00
WHH24_ATA	13	GCAATAATAACAAGAAGGGCA	AAGAAGTGACTGGTTTGGAG	NA	WHH1	94,38
WHH26_TAT	15	TTTCCAGAAGATGTGGTCAG	TACAAATCTCAGCACCACTC	NA	WHH1	98,06
WHH3_AAAGA	6	GAAGAAGTTTCCTCCTCTGG	GGTGGACTGAACCATTTCTT	NA	WHH1	98,92
WHH6_TTATT	7	AGGAGTTCTCAGTGATGAGA	AATACAGGCTCTGGGATAGT	378-404	WHH1	96,76
